# Patient-derived glioma organoids real time identification of IDH mutation, 1p/19q-codeletion and CDKN2A/B homozygous deletion with differential ion mobility spectrometry

**DOI:** 10.1007/s11060-024-04891-0

**Published:** 2024-11-23

**Authors:** Ismaïl Hermelo, Ilkka Haapala, Meri Mäkelä, Dafne Jacome Sanz, Anton Kontunen, Markus Karjalainen, Philipp Müller, Kai Lehtimäki, Matti Nykter, Juhana Frösén, Hannu Haapasalo, Antti Roine, Niku Oksala, Kristiina Nordfors, Antti Vehkaoja, Joonas Haapasalo

**Affiliations:** 1 Prostate Cancer Research Center, Faculty of Medicine and Health Technology, Tampere University and Tampere University Hospital, Tampere, Finland; 2 Department of Neurosurgery and Tays Cancer Center, Tampere University Hospital and Tampere University, Tampere, Finland; 3 Faculty of Medicine and Health Technology, Tampere University, Tampere, Finland; 4 Olfactomics Ltd., Tampere, Finland; 5 Department of Pediatric Hematology and Oncology and Tays Cancer Center, Tampere University Hospital, Tampere, Finland; 6 Fimlab Laboratories Ltd., Tampere, Finland; 7 Centre for Vascular Surgery and Interventional Radiology, Tampere University Hospital, Tampere, Finland; 8 Tampere Center for Child, Adolescent, and Maternal Health Research, Faculty of Medicine and Health Technology, Tampere University, Tampere, Finland

**Keywords:** CDKN2A/B homozygous loss, Differential mobility spectrometry, IDH mutation, Linear discriminant analysis, Patient-derived glioma organoids, 1p/19 co-deletion

## Abstract

**Purpose:**

Extent of brain tumor resection continues to be one of the central decisions taken during standard of care in glioma patients. Here, we aimed to evaluate the most essential molecular factors, such as IDH (isocitrate dehydrogenase) mutation in gliomas classification with patient-derived glioma organoids (PGOs) using differential mobility spectrometry (DMS).

**Methods:**

we prospectively recruited 12 glioma patients, 6 IDH-mutated and 6 IDH wild-type tumors, from which PGOs were generated *ex-vivo*. Altogether, 320 PGOs DMS spectra were analyzed with a classifier algorithm based on linear discriminant analysis (LDA).

**Results:**

LDA model classification accuracy (CA) obtained between IDH-mutant and IDH wild-type PGOs was 90% (91% sensitivity and 89% specificity). Furthermore, 1p/19q codeletion classification within IDH mutant PGOs reached 98% CA (93% sensitivity and 99% specificity), while CDKN2A/B homozygous loss status had 86% CA (63% sensitivity 93% specificity).

**Conclusion:**

DMS suitability to differentiate IDH-mutated PGOs was thus validated in ex vivo cultured samples, PGOs. Preliminary results regarding 1p/19q codeleted PGOs and CDKN2A/B loss PGOs identification endorse testing in a prospective intraoperative glioma patient cohort. Our results reveal a sample classification set-up that is compatible with real-time intraoperative surgery guidance.

**Supplementary Information:**

The online version contains supplementary material available at 10.1007/s11060-024-04891-0.

## Introduction

Brain tumors represent one of the major societal burdens, while new cases of malignant gliomas bearing worse prognosis are estimated to continue increasing [1]. For nearly two decades, standard of care has been based on temozolomide (TMZ) and radiation therapy, whilst therapeutic breakthroughs are still pending [2]. Conversely, brain tumor diagnostics underwent revisions to incorporate genetic phenotyping (i.e., molecular markers) alongside histopathology when identifying tumor lesions, among which the most notable are: the mutation of isocitrate dehydrogenase (IDH) enzyme, and codeletion of chromosome arms 1p and 19q (1p/19q codeletion) [3].

IDH enzymes comprise two subcellular differently located enzymes with same function (IDH1 and IDH2), both performing oxidative decarboxylation of isocitrate to form a-ketoglutarate (aKG) leading to, when mutated, the production of the oncometabolite 2-Hydroxyglutarate (2-HG) [3, 4]. Furthermore, even though IDH mutation is not restricted to a specific tumor histology, prognosis of glioma patients bearing IDH mutated tumors is generally more favorable than with IDH wild type glioma tumors [5, 6].

Moreover, within IDH mutant glioma tumors, 1p/19q codeletion is a genetic hallmark for oligodendrogliomas, with best overall survival [7]. The loss of 1p/19q results in differential transcriptome profiles from the genes located in chromosome 1p/19q compared to non-codeleted gliomas, likely indicative of genes related with better survival [8]. In terms of adjuvant therapy, oligodendrogliomas are more sensitive to chemotherapy with alkylating agents (e.g., TMZ) than other gliomas [9]. Germane to these genetic alterations, if harboring cyclin dependent kinase inhibitor 2A/B (CDKN2A/B) homozygous deletion, the impact on prognosis of the IDH mutated gliomas equals that of World Health Organization (WHO) grade 4 tumors rendering it of pivotal relevance [10]. Similarly, low protein expression levels of p16 (CDKN2A) were also predictive of poorer patient survival in oligodendrogliomas, reported over two decades ago [11].

Consequently, establishing tumor diagnosis bears paramount importance for accurate patient prognosis [12]. Yet, integration of histological tumor typing and grading with molecular markers faces the nature of glioma tumors: infiltration into brain parenchyma. For this reason, ensuring sufficient tumor tissue is available for diagnosis strengthens classification, leading to trustworthy prognosis. Furthermore, histopathological annotation together with genetic phenotyping contribute to delineate the treatment. However, such information becomes available completely post-surgery, and even though decision-making during surgery could evidently benefit from intraoperative assessment, currently there is no established method for biopsies near real-time identification.

Several adjuvant technological, and methodological strategies are being considered. Among others, fluorescence guided surgery, intraoperative image guidance and experimental techniques like mass spectrometry (MS) has recently been reported to robustly classify samples within 2 min [13], and analysis of diathermy smoke using differential mobility spectrometry (DMS) provide varied sample classification accuracies [14]. Needs for the sample preparation, measurement time, output data analysis and financial accessibility, influence how suitable the method might be for the intraoperative setting and its potential for universal implementation.

Even though DMS does not provide discrete information about sample components, the dispersion spectra provided by DMS serves as sample fingerprint, which can be used for characterization and identification of sample class. In our prior studies, retrospective and prospective ex vivo patient samples were tested with promising results using DMS-based classification [15–17]. Other reported methods include digital polymerase chain reaction (PCR) with over 5 h turnaround time [18], or immune-histochemistry based CDKN2A codeletion assessment (over 3 h time) [19], whilst real time PCR based method takes 1 h [20]. Alternative methods using methylation profiles (nanopore sequencing), obtained 72% diagnose accuracy within 90 min [21] and similar 89% accuracy in another study (ranging from 91 to 161 min) [22], which contrasts to DMS approach nearing real-time settings.

Despite our prior promising results with DMS-based classification of brain tumors, representative glioma models were considered necessary for performing a complementary proof-of-concept to our previous studies [15, 16]. For instance, prior projects included frozen samples (−70 °C) [15–17] while intraoperative assessment will be based on tissue biopsies analyzed on site, with minimal processing. Therefore, DMS-based assessment with cultured ex vivo tumors was arguably pertinent and closer to the tumor biopsy setting despite not being fresh tumor samples. To meet this end, single measurement session and generating ex vivo tumor organoid models was considered appropriate. Within gliomas, higher grade (WHO grade 4) primary cell monolayer-models from glioblastoma tumors have proven to be an invaluable resource [23], yet their transcriptomic and genomic changes have been also acknowledged [24]. Tumors in situ consist of neoplastic cells with varying states. Furthermore, neoplastic compartment within tumor microenvironment includes immune and other stromal cells, referred to as tumor immune microenvironment (TiME). Patient-derived glioma organoids (PGOs) retain TiME populations such as bone marrow-derived macrophages and microglia [25, 26] thus preserving sample-specific features, even though the PGO’s major component is neoplastic. Aiding to model *in-situ* tumor resection margins for DMS-spectra classification. Related to this, PGOs developed were exempt of necrotic tissue, assuring viable cell-communities, generating 3-dimensional organoids that preserved tumor’s genetic phenotype. Moreover, in our prior studies samples were laser-incised four times [15–17] without introducing bias, yet we opted to streamline processing time and maximize measurement consistency across samples.

We aimed to validate IDH mutation discernment based on DMS using samples on a biological state closer to intraoperative tumor biopsy, using patient-derived tumor organoid models and single measurements. Furthermore, as proof-of-concept, we studied whether DMS could further characterize IDH mutant gliomas of genetic prognostic markers 1p/19q codeletion and CDKN2A/B homozygous deletion status using PGOs.

## Methods

### Patient samples

A prospective cohort of 13 patients were recruited according to the magnetic resonance imaging appearance, and when indicative of diffuse glioma served as main patient inclusion criteria. Only one patient was excluded, after performing the final diagnosis and found to be pleomorphic xanthoastrocytoma. Patient-derived glioma organoids (PGOs) were established from human brain tumor samples from 12 patients undergoing gross total resection surgeries in Tampere University Hospital, between 2022 and 2023. Protocol followed [23] with herein modifications: after necrotic and hemorrhagic tissue removal, manual dissociation excluding enzymatic digestion and transferred onto uncoated dishes (Primaria, Corning) with conditions,: Neurobasal and DMEM:F12 (Thermo Fisher Scientific) (1:1 mix), with 1XB27 w/o vitamin A (Thermo Fisher Scientific) 1X and N2 supplements (Thermo Fisher Scientific), with 1X NEAAs (Thermo Fisher Scientific), 1X PenStrep (Thermo Fisher Scientific) and 1X GlutaMax (Thermo Fisher Scientific), including and human recombinant EGF and FGF2 (10 ng/ml, PEPROTECH, Thermo Fisher Scientific) [23] and insulin (2.5 μg/ml, Sigma-Aldrich) placed at 37 °C, 5% CO2, and 90% humidity sterile incubator. 2 to 4 days for medium replacement.

### Biological material and preservation method

**Fig. 1:**
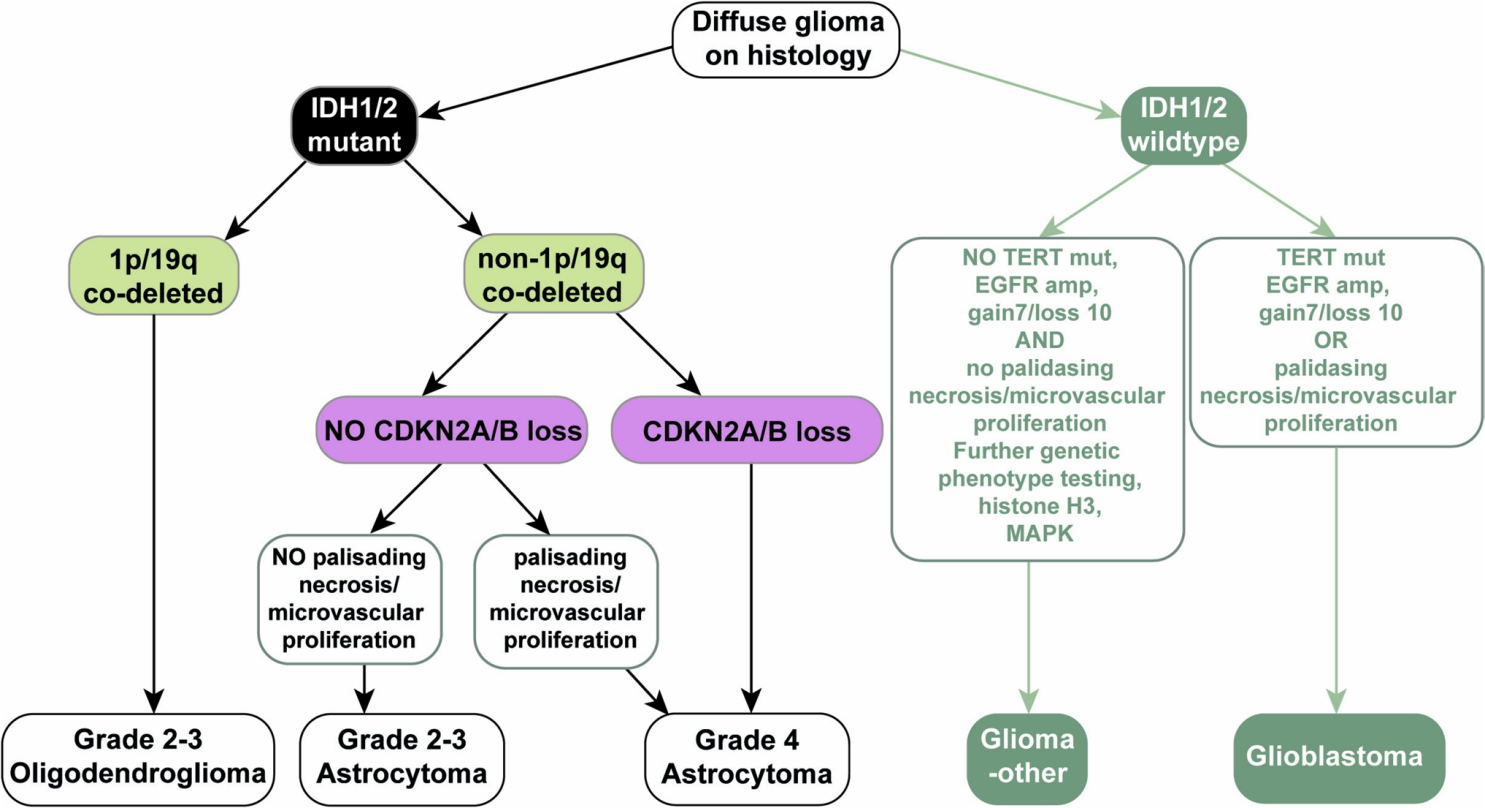
Flow diagram describing the role of 1p/19q co-deletion and CDKN2A/B homozygous deletion (loss) in the classification of IDH1/2 mutated tumors. Genetic features for IDH wild type tumors classification included. Diagram adapted from [43]; gain7/loss 10 stands for gain of chromosome 7 and loss of chromosome 10; isocitrate dehydrogenase 1/2, IDH1/2; reverse transcriptase, TERT; epidermal growth factor receptor, EGFR; mitogen-activated protein kinase, MAPK

**Fig. 2:**
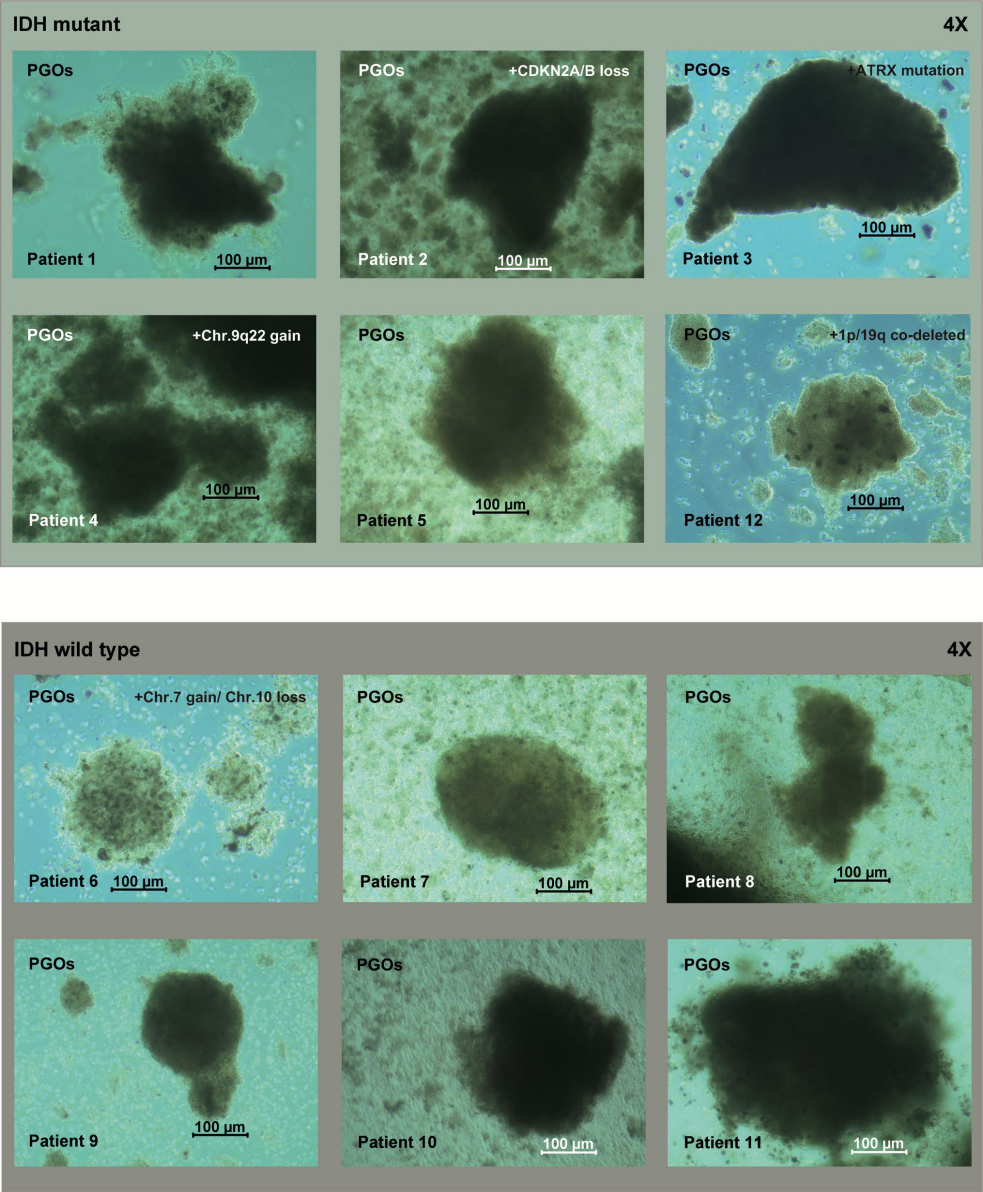
PGOs propagation ex-vivo. Bright field pictures (4X) of patient-derived organoids (PGOs) after 2 weeks of culture, showing varying sizes. Overall, higher grade gliomas expanded faster ex vivo. Upper panel IDH mutant PGOs and on lower panel IDH wild type PGOs. Scale bar indicates 100 µm

Sites used for diagnostic (see Fig. 1) biopsies and ex vivo cultures were adjacent (the tumor was divided as: formalin fixation, fresh-freezing, and culture samples). To preserve tumor-immune microenvironment, PGO were cultured in suspension for two weeks maximum (see Fig. 2) and stored in culturing media with DMSO 10% on liquid nitrogen −196 °C. Shorter culturing time ensured tumor’s genetic phenotype preservation.

The sample cohort comprised IDH-mutant (IDH-mut) grade 2–3 & grade 4 and IDH wild-type (IDH-wt) grade 4 tumors. As an integrated diagnosis protocol, morphological diagnostic assessment of tumor samples was obtained from an experienced neuropathologist. The samples were also processed to be scanned with virtual microscopy, slides which were also used for diagnosis.

### Clinical neuropathological sample characterization

Among IDH-mut samples [27], patients 3 and 7 had no other distinctive molecular alterations, patient 4 was also CDKN2A/B co-deleted, patient 5 had ATRX mutation, patient 6 had chromosome 9q22 gain and patient 14 had 1p19q-codeletion. Fluorescence in situ hybridization (FISH) analysis of CDKN2A/B and 1p19q loss were performed following principles of our previous study [28]. Among IDH-wt samples, following the similar approach patient 8 had chromosome 7 gain and chromosome 10 loss. Patient 9 was IDH-mut negative with no other distinctive alterations. For patients 8, 10, 11 and 12, histone H3 H3K27M mutation status was analyzed and determined as wild type (see Table 1). Samples negative for IDH mutation immunohistochemistry staining were subjected to PCR testing (Supplementary Table 1).

**Table 1:** Histological an integrated diagnosis

Patient cohort						Patient-derived PGOs samples		
Patient ID	Neuropathological features	Final diagnostic	WHO grade	Sex	Age	PGOs seeded	PGOs measured	PGOs missed
Patient 1	IDH-mutant, non 1p/19q -codeleted	Astrocytoma, IDH-mutant	3	M	26–30	32	26	6 (19%)
Patient 2	IDH-mutant, CDKN2A/B co-deleted	Astrocytoma, IDH-mutant	4	M	46–50	32	32	–
Patient 3	IDH-mutant, ATRX mutant, non 1p/19q co-deleted	Astrocytoma, IDH-mutant	2	F	31–35	32	24	8 (25%)
Patient 4	IDH -mutant, no CDKN2A/B co-deletion, Chr. 9 q22 gain	Astrocytoma, IDH-mutant	4	M	36–40	32	25	7 (22%)
Patient 5	IDH -mutant, non 1p/19q -co-deleted, no CDKN2A/B co-deletion	Astrocytoma, IDH-mutant	2	F	51–55	32	31	1 (3%)
Patient 6	IDH/H3 wild type, Chr. 7 gain/ Chr. 10 loss	Glioblastoma, IDH wild type	4	M	46–50	16	12	4 (25%)
Patient 7	IDH wild type, no CDKN2A/B co-deletion	Glioblastoma, IDH wild type	4	M	46–50	32	30	2 (6%)
Patient 8	IDH/H3 wild type	Glioblastoma, IDH wild type	4	M	81–85	32	30	2 (6%)
Patient 9	IDH/H3 wild type	Glioblastoma, IDH wild type	4	M	61–65	24	21	3 (12%)
Patient 10	IDH/H3 wild type	Glioblastoma, IDH wild type	4	M	41–45	32	32	–
Patient 11	IDH wild type	Glioblastoma, IDH wild type	4	M	51–55	32	30	2 (6%)
Patient 12	IDH-mutant, 1p/19q co-deleted	Oligodendroglioma, IDH-mutant, 1p/19q co-deleted	3	F	41–45	24	14	10 (42%)

Cohort of patient samples used in experimental procedure, based on World Health Organization (WHO) classification 5th edition 2021 [42]; PGOs, Patient-derived glioma organoids; PGOs missed: percentage of PGOs not measured due to missed laser burn; IDH, isocitrate dehydrogenase; Patient tumor biopsies were evaluated by neuropathologist diagnosis (Diagnostic column) during 2022–2023; patient-derived organoid, PGO; isocitrate dehydrogenase, IDH; chromosome arms 1p and 19q co-deletion, 1p/19q co-deleted; cyclin dependent kinase inhibitor 2A/B, CDKN2A/B; H3, histone H3 H3K27M mutation status; e.g. Chr. 7 gain, chromosome 7 gain; M, male; F, female

Epidermal growth factor receptor (EGFR) amplification: EGFR gene amplification was assessed with chromogenic in situ hybridization, which included EGFR probe detection with mouse antidigoxigenin (diluted 1:300; Roche Biochemicals, Mannheim, Germany), and Powervision + immunoperoxidase kit reagents (Immunovision Inc., Daly City, CA, USA). A non-amplified gene copy number was defined as one to five signals per nucleus. Amplification was defined as six or more signals per nucleus in over 50% of cancer cells, as described previously [29]. Telomerase reverse transcriptase (TERT) promoter region mutation: SNaPshot analysis was performed for analysis of 3 mutational hot spots: C228, C242, and C250 (chr5:1,295,228C > T; chr5:1,295,242–243CC > TT; chr5:1,295,250 C > T, respectively; hg19) in the TERT promoter as described previously [30, 31]. Chromosome 10 loss and 7 gain: to determine the DNA methylation status the Infinium HumanMethylation450 Bead-Chip (450 k) array (Illumina, Carlsbad, California, USA) was used, covering 482,421 CpG sites according to the manufacturer’s instructions. Copy number profiles (CNP) were calculated from the methylation array data as previously described [32]. Amplifications in DNA copy number profile were defined as focal regions of copy number gain with a notably higher amplitude compared to regions of suspected single-copy gains [33, 34].

## PGOs analysis setup

**Fig. 3:**
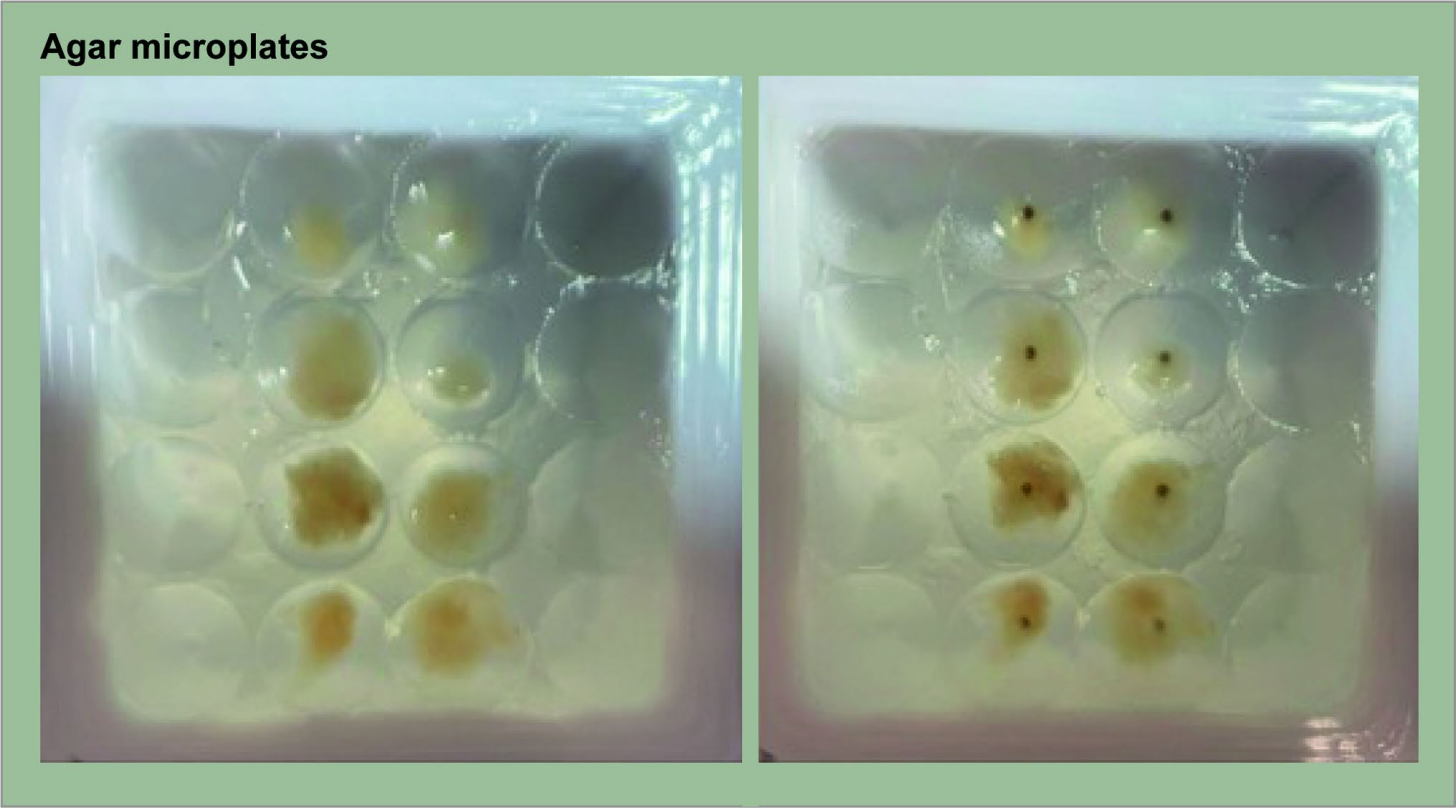
Microplates with PGOs for sampling. Example of sample plate before (left) and after (right) sampling. Sample plate 12.3; as clearly shows, each patient-derived glioma organoid (PGO) was successfully sampled with the laser

The measurement system has been thoroughly described in [15, 16]. Main components of the system are humidifier, sampling unit, and DMS sensor analyzer. Samples were placed on plastic microplates containing 0.18 mL agar, molded with 3D-printed resin prompt to generate 16 well agar-microplates (see Fig. 3), with 8 wells PGOs seeded, and remaining left for blank measurements. To ensure sample viability, all PGOs were seeded and covered with parafilm overnight at 4 °C before the measurement session. Simultaneous seeding ensured using non-deteriorated PGOs and a single measurement session.

### DMS analysis

We previously investigated the applicability of DMS for brain tumor classification in different study settings [15, 16]. DMS is an advanced adaptation of ion mobility spectrometry (IMS). IMS is a modality that characterizes substances based on the mobility differences of their gaseous ion swarms. In DMS, however, ion mobility differences relating to electric field strength are measured within a high-field asymmetric waveform, in which ions cluster with the drift of gas molecules resulting in different trajectories between low and high electric fields. This enhances the separability of ions that have similar mobilities in a constant low electric field [35]. Results of measured detector responses are presented as a data matrix, or dispersion spectrum, which is sample-specific.

DMS used was the commercial IonVision instrument by Olfactomics Ltd (Tampere, Finland). Ions are produced with 4.9 keV soft X-ray photoionization and dispersed in a 1 MHz electric field with a duty cycle of 22%. Each sample was incised once with custom-built CO_2_ laser evaporator. Laser system was computer-controlled, and each incision consisted of 475 pulses, each pulse taking 2 ms with 33 ms pauses in between. 30 s pauses were kept after each incision to allow the system to clear contamination between samples. Length of single incision was 16.625 s, and DMS sampling time was matched by adjusting spectrum resolution. To ensure sample consistency 5 s waiting time from incision to start of a DMS measurement was used. DMS measurement parameters were compensation voltage (Ucv) −2 to 10 V with 60 increments; separation voltage (Usv) 200–1000 V with 40 increments, resulting in currents being measured for 2400 different combinations of compensation and separation voltages. Bias voltage of detector was −6 V.

### Data analysis and statistical methods

**Fig. 4:**
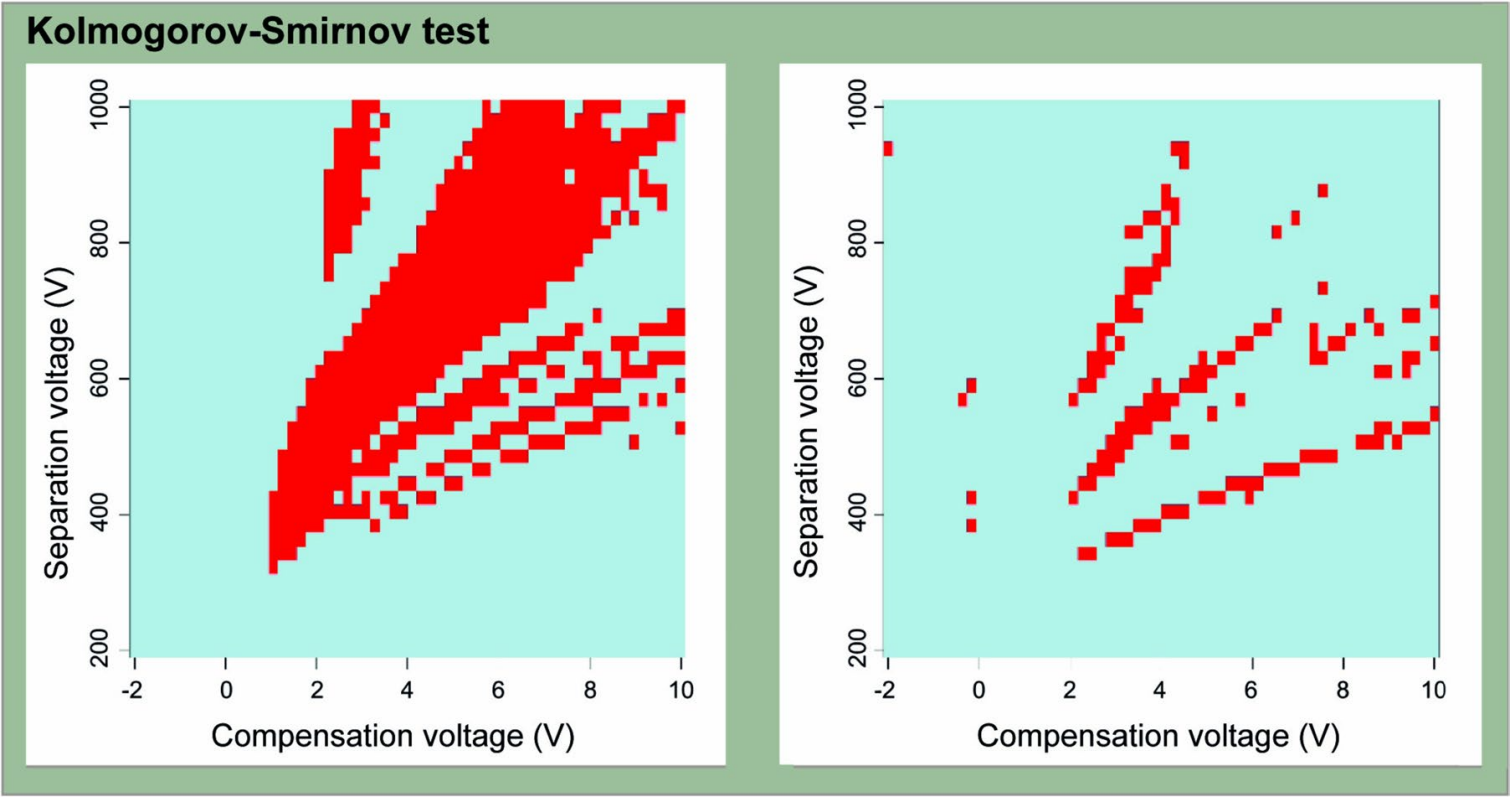
Significantly varying areas of the DMS-spectra between IDH-mut and IDH-wt samples. Several peaks were identified as significantly different between IDH mutant (IDH-mut) and IDH-wild type (IDH-wt) PGO groups dispersion plot spectra according to the Kolmogorov–Smirnov (KS)-test. The original significance level (alpha) was set to 0.05, and after the Bonferroni correction the level was set approximately to 10^–5^, so that statistically different points can be located. Pixels in red are tagged as significantly different at corrected significance level between the two PGOs groups, and light blue points indicate no statistical difference. Positive ion side on the left and negative ion side on the right panel. Positive ion side drawn on the left panel indicating regions in where spectra between IDH-mut and IDH-wt PGOs differ (red pixels). Similarly, negative ion spectra on the right graph showing points of KS-test significant differences. Both sides were used in the linear discriminant analysis classification

Each sampled PGO resulted in one DMS-spectrum. Tumor classification was performed as two-class classification between sample groups. Leave-one-out cross-validated (LOOCV) linear discriminant analysis (LDA) models were utilized in Python. Singular value decomposition was used as the solver for LDA. In addition, Kolmogorov–Smirnov tests were performed between binary groups to discern the presence of statistically significant differences in spectra between groups (see Fig. 4). Significance level was set at 0.05 and adjusted with Bonferroni correction. Data was also pretreated with linear trend removal before the analysis.

In addition to LOOCV, leave-one-patient-out cross-validation was performed for IDH status comparison. This cross validation (CV) method ensured that train and test groups were independent. For error estimation, bootstrapping technique was utilized for all comparisons explored. Dataset was resampled 1000 times for each comparison. Original number of samples was resampled, and then resampled data was train/test split with a 70/30 partition to perform classification.

LDA classifier training was done on a separate day to laser measurements. In this study, the LOOCV in addition to classifications took 5 min.

## Results

The significance of most relevant genetic alterations for tumor classification and their effect on patient prognosis are summarized in Fig. 1. Accordingly, the objectives of the analysis were itemized in three aim categories and candidate variables: (1) to discern the presence of IDH mutation as main question for validation purposes, followed by (2) the presence of either 1p/19q codeletion or CDKN2A/B homozygous deletion within IDH mutant samples. And related to the second aim, the aim (3) was to preliminarily test the presence of CDKN2A/B homozygous deletion within IDH mutant samples.

### DMS classifies glioma tumors according to IDH mutation status

Following the two-class classifications for all comparisons, samples were classified based on presence of IDH mutation (aim 1) with leaving-one-out (i.e. single PGOs) cross-validated classifier. The classification accuracy (CA) reached 90% (90.2%, Table 2) with substantial sensitivity (91%) and specificity (89%), thus substantiating our prior results [15, 16]. This was further endorsed by DMS spectra ‘unique features found statistically significant with Kolmogorov–Smirnov (KS) test (Fig. 4) [15, 16]. Since multiple PGOs were tested per patient, intra-patient classification variability was also observed (Supplementary Fig. 1).

**Table 2:** LDA Two-class classification results obtained using leave-one PGO-out cross-validation

Binary classification between…	Accuracy (%)	Sensitivity (%)	Specificity (%)
IDH mutant (PGOs = 152) and IDH wild-type (PGOs = 155)	90.2	91	89
1p/19q co-deleted or CDKN2A/B co-deleted (PGOs = 46) and other IDH mutant samples (PGOs = 106)	82.2	70	88
CDKN2A/B co-deleted (PGOs = 32) and other IDH mutant samples (PGOs = 120)	86.2	63	93
1p/19q co-deleted (PGOs = 14) and other IDH mutant samples (PGOs = 138)	98.0	93	99

PGO, patient-derived glioma organoid; isocitrate dehydrogenase 1/2, IDH

**Fig. 5:**
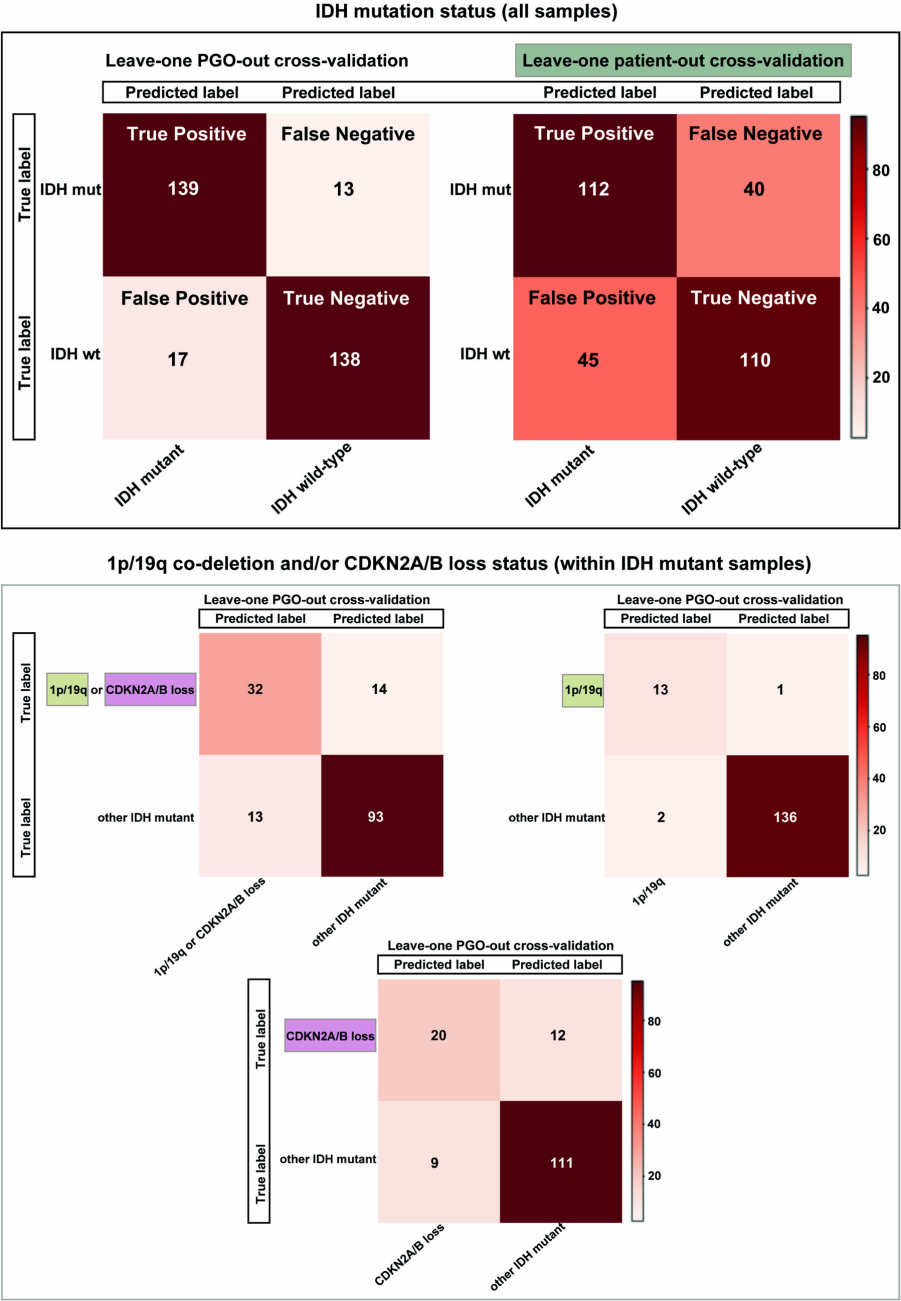
Confusion matrices for the performed two-class classification tests. Upper panel shows the results for IDH mutation status classification using leave-one-sample [PGOs]-out (left) and leave-one-patient-out (right) cross validation. Lower panel shows 1p/19q co-deletion or CDKN2A/B loss status (left), 1p/19q co-deletion status (right) or CDKN2A/B loss status (bottom) of IDH mutant PGOs. Confusion matrices for indicated sample groups and assigned categories by LDA classification method, including e.g. “all” PGOs IDH wild type and IDH mutant (upper panel), or within IDH mutant samples (lower panel) 1p/19q co-deletion and/or CDKN2A/B loss PGOs status classification. To avoid overfitting, leave-one PGO/ or patient [PGOs set]-out cross-validation as indicated. Correctly assigned PGOs in “True Positive” and “True Negative”, including PGOs counts across all labels (True and Predicted) for sample size and category balance evaluation; *LDA* linear discriminant analysis, *PGO* patient-derived glioma organoid

Regarding the leave-one-patient out cross-validation (LOOCV) (i.e. leaving out the entire PGOs set from a single patient), the CA reached 72% (72.3%, Table 3) with comparable sensitivity 74%, and specificity 71%, suggesting IDH status classification robustness (patients or PGOs number in Table 1, and classifier-based category predictions in Fig. 5). This was further endorsed by error estimation using resampling methods reaching 87% CA (78 to 94%, at 95% confidence interval; Table 4).

**Table 3:** LDA Two-class classification results obtained using leave-one-patient-out cross-validation

Binary classification between…	Accuracy (%)	Sensitivity (%)	Specificity (%)
IDH mutant (n = 6) and IDH wild-type (n = 6)	72.3	74	71

Patient-derived organoid, PGO; isocitrate dehydrogenase, IDH; chromosome arms 1p and 19q co-deletion, 1p/19q co-deleted; cyclin dependent kinase inhibitor 2A/B, CDKN2A/B; n, patient counts

**Table 4:** Error estimation

Comparison groups	Median [95% confidence interval]		
	Accuracy (%)	Sensitivity (%)	Specificity (%)
IDH mutant (PGOs = 152) and IDH wild-type (PGOs = 155)	87 [78, 94]	89 [76, 98]	85 [74, 96]
1p/19q co-deleted or CDKN2A/B co-deleted (PGOs = 46) and other IDH mutant samples (PGOs = 106)	80 [67, 89]	57 [29, 86]	91 [72, 100]
CDKN2A/B co-deleted (PGOs = 32) and other IDH mutant samples (PGOs = 120)	85 [74, 93]	50 [20, 90]	94 [81, 100]
1p/19q co-deleted (PGOs = 14) and other IDH mutant samples (PGOs = 138)	98 [91, 100]	100 [50, 100]	98 [90, 100]

Bootstrapping results from 1000 rounds of resampling. Main values are the median and numbers presented in brackets the 95% confidence interval; patient-derived organoid, PGO; isocitrate dehydrogenase, IDH; chromosome arms 1p and 19q co-deletion, 1p/19q co-deleted; cyclin dependent kinase inhibitor 2A/B, CDKN2A/B

### DMS detects 1p/19q codeletion and CDKN2A/B loss

Next, within IDH mutant samples, the feasibility to classify whether 1p/19q codeletion or CDKN2A/B homozygous deletion was present (aim 2), over 80% (82.2%) CA with (sensitivity 70%, specificity 88%) was achieved (with 46 vs 106 PGOs respectively, Fig. 5). Moreover, the discerning potential of 1p/19q codeletion within IDH mutant samples was tested, and CA 98% with sensitivity 93% and specificity 99% (14 vs 138 PGOs respectively, Fig. 5). In addition, enquiring the CDKN2A/B codeletion among IDH mutant samples provided CA over 85% (86.2%) with respective (sensitivity 63%, specificity 93%, Table 4) with (32 vs 120 PGOs) sized groups.

## Discussion

The obtained results endorse DMS technology for swift IDH status classification (CA 90.2%). With a pre-trained LDA classifier, the classification result will be obtained in 4 s, therefore from start of DMS-measurement to LDA classifier prediction entailing less than 21 s nearing real-time. Tumor classifications are likely due to differences in metabolic molecules between IDH mutant and wild type tumors [13, 16]. Furthermore, these results suggest potential for classification beyond IDH mutation, namely prognostic molecular markers, 1p/19q codeletion and CDKN2A/B codeletion [3, 6, 10, 36, 37]. With our set-up and patient cohort, 1p/19q codeletion classification within IDH mutant PGOs had notable sensitivity (93%) and specificity (99%), indicating suitability for intraoperative surgery guidance, based on tumor biopsy single laser incision measurement. Even though CDKN2A/B loss classification did not had comparable CA as for 1p/19q codeletion (86.2% vs 98.9% respectively), specificity was 93% (correctly assigning CDKN2A/B homozygous deletion negative PGOs), thus discriminating real CDKN2A/B loss negative PGOs, equitably relevant prognosis wise [10, 37]. However, CA regarding 1p/19q co-deletion and CDKN2A/B loss molecular markers within IDH mutant PGOs may be noted prudently, as the class sizes were imbalanced.

Developing PGOs was based on studies reporting the preservation of some of the lesion’s intrinsic features, such as the hypoxic gradients that included neoplastic and stromal cell–cell interactions [25]. Ex-vivo cultured PGOs ensured that part of the tumor’s TiME was present when performing DMS measurements. Furthermore, since PGOs establishment efficiency was 100% regardless of patient’s genetic phenotype, 152 PGOs derived from 6 IDH mutated tumors were studied along with 168 PGOs from 6 IDH wild type tumors. However, it should be noted that PGOs-based results should be taken as preliminary results. PGOs cannot be considered equivalent to fresh tumor samples and formal validation of the DMS classification approach will be obtained using fresh living tumor samples under intraoperative settings.

Moreover, IDH mutation and 1p/19q codeletion status are conjointly decisive on treatment decisions guidance [12, 37]. Accordingly, patients with IDH mutated or IDH wild type tumors benefit from gross-total surgical resection (GTR) and at the same time the aim to minimize neurological sequelae is also considered [36, 38, 39]. However, in spite of the importance of GTR, patients with IDH mutated tumors comparatively gain higher survival benefit from maximal resection, favoring aggressive resection strategy. Contrastingly, GTR compared with nearly total resection of the tumor seems to be less crucial for the prognosis of oligodendrogliomas than for IDH mutant non-1p19q codeleted gliomas [40]. Patient prognosis for IDH wild type tumors is worse regardless of the treatment modality, thus avoiding causing neurological deficits becomes an essential deliberation.

DMS technology provides brain tumor’s rapid characterization as a cost-effective opportunity and emerging adjuvant methodology [15–17]. Our results point to the feasibility for detection of IDH mutation and 1p19q deletion status intraoperatively. Furthermore, the IDH mutation has also issued novel targeted therapeutic avenues, like the IDH enzymes dual inhibitor vorasidenib in IDH mutant glioma patients (double-blind phase 3 clinical trial). Thus, underscoring the importance of IDH mutation intraoperative detection for clinical decision making [41]. Similarly, our method could aid in classifying CDKN2A/B-homozygous loss negative samples when proven on a larger cohort, and since loss of CDKN2A/B in IDH mutant astrocytoma is the principal biomarker for poor prognosis, we propose DMS’ potential for its rapid identification of patients with poorer prognosis, hence amenable to undergo GTR.

Besides, the DMS method shows capabilities to assist neuropathology decision making, regarding diagnostic and prognostic biomarkers. Testing IDH wild type gliomas if bearing EGFR amplification, TERT promoter region mutation, or chromosome 7 gain and chromosome 10 loss genetic alterations versus other gliomas, could be similarly used to classify glioblastoma (WHO classification 5th edition 2021) [12, 42] samples. As presented here, before the intraoperative settings it could be tested using our PGOs-based approach in future.

### Limitations of the study

Even though PGOs propagation provided replicates of the tumor sample, the number of patients and especially, the number of different tumor subtypes, is limited. Since this is a prospective study based on PGOs for which tumor diagnostics were not available a priori, CDKN2A/B loss and 1p/19q co-deletion groups were non-balanced. Nonetheless, noting IDH mutant brain tumor’s incidence and that this is a single institution prospective study, we successfully established PGOs from all recruited patients and conducted the study in a proof-of-concept set-up. Accordingly, since PGOs served as an intraoperative biopsy proxy, we don’t postulate PGOs generation as part of the DMS-based classification pipeline. The goal is to perform a single laser-incision to the tumor biopsies obtained during surgery, which mirrors several PGOs per patient, in where intraoperative setting will include several tumor sampling points for the same patient. The PGOs approach therefore closely models the real time set-up (from laser incision to LDA classification below 30 s).

Moreover, considering the number of patients in this study, PGOs sample cohorts were used during the LOOCV training rounds. Albeit sample classification potential is shown, to upgrade to patient classification, a larger patient cohort (of herein studied genetic phenotypes) is needed to further train the classifier, on additional new patients.

## Conclusion

Our results further validate DMS in discriminating IDH mutant from IDH wild type samples using PGOs models (as biopsy surrogates) with single measurements, the first of its kind. Furthermore, 1p/19q co-deletion and CDKN2A/B loss classification potential is reported. Hence, we propose next to test DMS-based tumor type identification on a larger glioma patient prospective cohort intraoperatively.

## Supplementary Information

Below is the link to the electronic supplementary material.

Supplementary file1 (TIF 20263 KB) Supplementary Fig. 1 DMS-based IDH status prediction across individual patient PGOs. Multiple PGOs IDH status prediction suggests the presence of intra-patient classification variability. Upper (IDH mutant) and lower (IDH wild type) panel show the results of LDA classifier predictions per PGO e.g. from patient 1, 21 PGOs were analyzed with DMS (column tiles) and the actual status (grey) together with classifier predictions: shown at patient (black) and at PGO level (green or purple) to visualize in IDH calling among different organoids within patient

Supplementary file2 (DOCX 28 KB)

## Data Availability

No datasets were generated or analysed during the current study.

## Abbreviations

IDH: Isocitrate dehydrogenase
PGOs: Patient-derived glioma organoids
DMS: Differential mobility spectrometry
LDA: Linear discriminant analysis
CA: Classification accuracy
aKG: A-ketoglutarate
1p/19q codeletion: Chromosome arms 1p and 19q
CDKN2A/B: Cyclin dependent kinase inhibitor 2A/B
WHO: World health organization
MS: Mass spectrometry
PCR: Polymerase chain reaction
TiME: Tumor immune microenvironment
FISH: Fluorescence in situ hybridization
LOOCV: Leave-one-out cross-validated
KS test: Kolmogorov–Smirnov tests
CV: Cross validation
