# Supplementary figures and images for: Patient-derived glioma organoids real time identification of IDH mutation, 1p/19q-codeletion and CDKN2A/B homozygous deletion with differential ion mobility spectrometry
Source: J Neurooncol. 2024 Nov 23;171(3):691–703. doi: 10.1007/s11060-024-04891-0 (PMC11729090; doi:10.1007/s11060-024-04891-0)

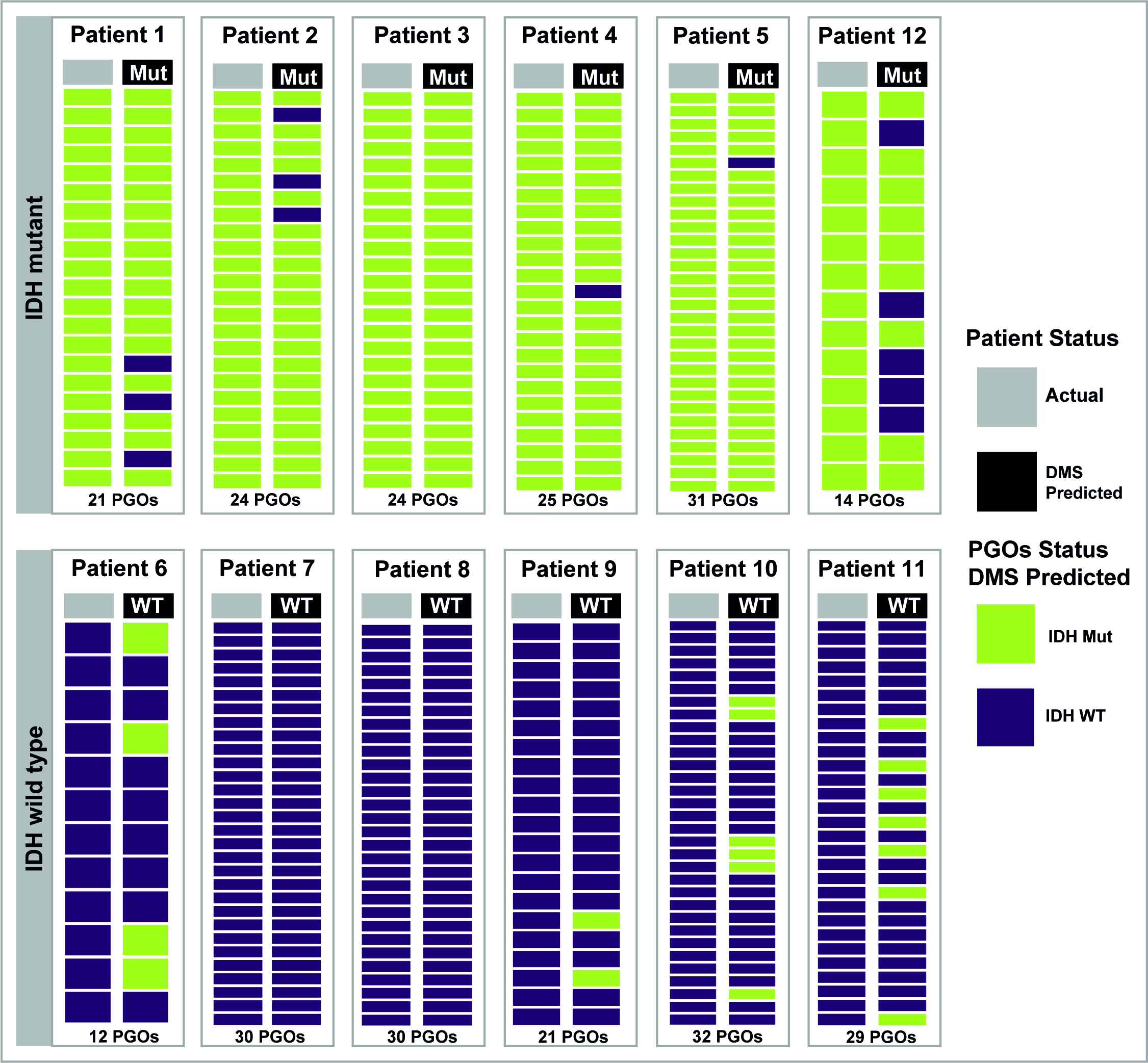

Supplement: Supplementary file 1 — Supplementary file1 (TIF 20263 KB) Supplementary Fig. 1 DMS-based IDH status prediction across individual patient PGOs. Multiple PGOs IDH status prediction suggests the presence of intra-patient classification variability. Upper (IDH mutant) and lower (IDH wild type) panel show the results of LDA classifier predictions per PGO e.g. from patient 1, 21 PGOs were analyzed with DMS (column tiles) and the actual status (grey) together with classifier predictions: shown at patient (black) and at PGO level (green or purple) to visualize in IDH calling among different organoids within patient [file 11060_2024_4891_MOESM1_ESM.tif]
