# Supplementary material for: Patient-derived glioma organoids real time identification of IDH mutation, 1p/19q-codeletion and CDKN2A/B homozygous deletion with differential ion mobility spectrometry
Source: J Neurooncol. 2024 Nov 23;171(3):691–703. doi: 10.1007/s11060-024-04891-0 (PMC11729090; doi:10.1007/s11060-024-04891-0)
Supplement: Supplementary file 2 — Supplementary file2 (DOCX 28 KB) [file 11060_2024_4891_MOESM2_ESM.docx]

| Patient Cohort | | | |
| --- | --- | --- | --- |
| Patient ID | IDH1 IHC | PCR | Age |
| **Patient 1** | Negative | IDH1 pos | 26-30 |
| **Patient 2** | Negative | IDH1 pos | 46-50 |
| **Patient 3** | Positive | Not performed | 31-35 |
| **Patient 4** | Positive | Not performed | 36-40 |
| **Patient 5** | Positive | Not performed | 51-55 |
| **Patient 6** | Negative | IDH1 and IDH2 neg | 46-50 |
| **Patient 7** | Negative | IDH1 and IDH2 neg | 46-50 |
| **Patient 8** | Negative | Not performed | 81-85 |
| **Patient 9** | Negative | IDH1 and IDH2 neg | 61-65 |
| **Patient 10** | Negative | IDH1 and IDH2 neg | 41-45 |
| **Patient 11** | Negative | IDH1 and IDH2 neg | 51-55 |
| **Patient 12** | Positive | Not performed | 41-45 |

**Supplementary Table 1.-polymerase chain reaction (PCR)-based assessment of IDH status.**

Results from the IHC staining for IDH1(R132) and PCR tests (when applicable) on parental tumors. IHC, immunohistochemistry; IDH1 or -2, isocitrate dehydrogenase 1 or 2; pos, positive; neg, negative.
